# Efficacy of Mesenchymal Stem Cells in Suppression of Hepatocarcinorigenesis in Rats: Possible Role of Wnt Signaling

**DOI:** 10.1186/1756-9966-30-49

**Published:** 2011-05-05

**Authors:** Mohamed T Abdel aziz, Mohamed F El Asmar, Hazem M Atta, Soheir Mahfouz, Hanan H Fouad, Nagwa K Roshdy, Laila A Rashed, Dina Sabry, Amira A Hassouna, Fatma M Taha

**Affiliations:** 1 Unit of Biochemistry and Molecular Biology (UBMB), Department of Medical Biochemistry, Faculty of Medicine, Cairo University, Cairo, Egypt; 2 Department of Medical Biochemistry, Faculty of Medicine, Ain Shams University, Cairo, Egypt; 3 Department of Pathology, Faculty of Medicine, Cairo University, Cairo, Egypt

## Abstract

**Background:**

The present study was conducted to evaluate the tumor suppressive effects of bone marrow derived mesenchymal stem cells (MSCs) in an experimental hepatocellular carcinoma (HCC) model in rats and to investigate the possible role of Wnt signaling in hepato-carcinogenesis.

**Methods:**

Ninety rats were included in the study and were divided equally into: Control group, rats which received MSCs only, rats which received MSCs vehicle only, HCC group induced by diethylnitroseamine (DENA) and CCl_**4**_, rats which received MSCs after HCC induction, rats which received MSCs before HCC induction. Histopathological examination and gene expression of Wnt signaling target genes by real time, reverse transcription-polymerase chain reaction (RT-PCR) in rat liver tissue, in addition to serum levels of ALT, AST and alpha fetoprotein were performed in all groups.

**Results:**

Histopathological examination of liver tissue from animals which received DENA-CCl_4 _only, revealed the presence of anaplastic carcinoma cells and macro-regenerative nodules type II with foci of large and small cell dysplasia. Administration of MSCs into rats after induction of experimental HCC improved the histopathological picture which showed minimal liver cell damage, reversible changes, areas of cell drop out filled with stem cells. Gene expression in rat liver tissue demonstrated that MSCs downregulated *β-catenin*, proliferating cell nuclear antigen (*PCNA*), *cyclin D *and *survivin *genes expression in liver tissues after HCC induction. Amelioration of the liver status after administration of MSCs has been inferred by the significant decrease of ALT, AST and Alpha fetoprotein serum levels. Administration of MSCs before HCC induction did not show any tumor suppressive or protective effect.

**Conclusions:**

Administration of MSCs in chemically induced HCC has tumor suppressive effects as evidenced by down regulation of Wnt signaling target genes concerned with antiapoptosis, mitogenesis, cell proliferation and cell cycle regulation, with subsequent amelioration of liver histopathological picture and liver function.

## Background

Hepatocellular carcinoma (HCC) is a highly prevalent, treatment-resistant malignancy with a multifaceted molecular pathogenesis[1]. It is a significant worldwide health problem with as many as 500,000 new cases diagnosed each year[2]. In Egypt, HCC is third among cancers in men with >8000 new cases predicted by 2012[3]. Current evidence indicates that during hepatocarcinogenesis, two main pathogenic mechanisms prevail: cirrhosis associated with hepatic regeneration after tissue damage and mutations occurring in oncogenes or tumor suppressor genes. Both mechanisms have been linked with alterations in several important cellular signaling pathways. These pathways are of interest from a therapeutic perspective, because targeting them may help to reverse, delay or prevent tumorigenesis[1]. In experimental animals interferon-α (*IFN-α*) gene therapy exerts significant protective effects, but more so when the gene is administered before fibrogenic and carcinogenic induction in hepatic tissues[4]. In humans, in the absence of any antiviral response, a course of interferon alpha does not reduce the risks of liver cancer or liver failure[5]. Whereas, after curative treatment of primary tumour; IFN-alpha therapy may be effective for the prevention of HCC recurrence[6]. Therefore providing new therapeutic modalities may provide a better way for treatment of HCC and amelioration of tumor mass prior to surgical intervention.

Advances in stem cell biology have made the prospect of cell therapy and tissue regeneration a clinical reality[7]. In this rapidly expanding field of cell based therapy, more attention has been paid to the relationship between stem cells and tumor cells. Qiao and coworkers reported that human mesenchymal stem cells (hMSCs) can home to tumor sites and inhibit the growth of tumor cells[8]. Furthermore, the authors reported that hMSCs inhibit the malignant phenotypes of the H7402 and HepG2 human liver cancer cell lines [9]. The stem cell microenvironment has an essential role in preventing carcinogenesis by providing signals to inhibit proliferation and to promote differentiation [10]. Furthermore, tumor cells may secrete proteins that can activate signaling pathways which facilitate hMSC migration to the tumor site [11]. Moreover, MSCs not only support hematopoiesis, but also exhibit a profound immune-suppressive activity that targets mainly T-cell proliferation[12]. In an animal model of hepatic injury, the researchers suggested that MSCs might become a more suitable source for Stem Cell-based therapies than hepatic stem cells, because of their immunological properties as MSCs are less immunogenic and can induce tolerance upon transplantation[13]. Moreover, MSCs showed the highest potential for liver regeneration compared with other BM cell subpopulations [14].

Little is known about the underlying molecular mechanisms that link MSCs to the targeted inhibition of tumor cells. Despite their distinct origins, stem cells and tumor cells share many characteristics[15,16]. In particular, they have similar signaling pathways that regulate self-renewal and differentiation[17-20]. The Wnt signaling pathway has been widely investigated in recent years. It has an important role in stem cell self-renewal and differentiation, and aberrant activation of the Wnt signaling pathway has been implicated in human tumor progression[21]. This has raised the possibility that the tightly regulated self-renewal process that is mediated by Wnt signaling in stem cells and progenitor cells may be subverted in cancer cells to allow malignant proliferation. Wnt signaling regulates genes that are involved in cell metabolism, proliferation, cell-cycle regulation and apoptosis[22].

The present work aimed at evaluating the tumor suppressive effects of MSCs on the in vivo progression of HCC, and to investigate the possible role of Wnt signaling in tumor tissues by assessing the gene expression profile of some of the Wnt signaling target genes:*cyclin D, PCNA, survivin, β-catenin*.

## Methods

Ninety albino female rats inbred strain (Cux1: HEL1) of matched age and weight (6 months-1 year & 120-150 gm) were included in the study. Animals were inbred in the experimental animal unit, Faculty of Medicine, Cairo University. Rats were maintained according to the standard guidelines of Institutional Animal Care and Use Committee and after Institutional Review Board approval. Animals were fed a semi-purified diet that contained (gm/kg): 200 casein, 555 sucrose, 100 cellulose, 100 fat blends, 35 vitamin mix, and 35 mineral mix [23]. They were divided equally into the following groups:1^st ^control rats group, 2^nd ^group received MSCs only (3 × 10^**6 **^cells intravenously), 3^rd ^group received MSCs solvent, 4^th ^HCC group induced by diethyl-nitroseamine (DENA) and CCl_**4**_, 5^th ^group received MSCs after induction of HCC, 6^th ^group received MSCs before induction of HCC.

### Preparation of BM-derived MSCs

Bone marrow was harvested by flushing the tibiae and femurs of 6-week-old white albino male rats with Dulbecco's modified Eagle's medium (DMEM, GIBCO/BRL) supplemented with 10% fetal bovine serum (GIBCO/BRL). Nucleated cells were isolated with a density gradient [Ficoll/Paque (Pharmacia)] and resuspended in complete culture medium supplemented with 1% penicillin-streptomycin (GIBCO/BRL). Cells were incubated at 37°C in 5% humidified CO_**2 **_for 12-14 days as primary culture or upon formation of large colonies. When large colonies developed (80-90% confluence), cultures were washed twice with phosphate buffer saline (PBS) and the cells were trypsinized with 0.25% trypsin in 1 mM EDTA (GIBCO/BRL) for 5 min at 37°C. After centrifugation, cells were resuspended with serum-supplemented medium and incubated in 50 cm^2 ^culture flasks (Falcon). The resulting cultures were referred to as first-passage cultures[24]. On day 14, the adherent colonies of cells were trypsinized, and counted. Cells were identified as being MSCs by their morphology, adherence, and their power to differentiate into osteocytes[25] and chondrocytes[26]. Differentiation into osteocytes was achieved by adding 1-1000 nM dexamethasone, 0.25 mM ascorbic acid, and 1-10 mM beta-glycerophosphate to the medium. Differentiation of MSCs into osteoblasts was achieved through morphological changes, Alzarin red staining of differentiated osteoblasts and RT-PCR gene expression of osteonectin in differentiated cells. Differentiation into chondrocyte was achieved by adding 500 ng/mL bone morphogenetic protein-2 (BMP-2; R&D Systems, USA) and 10 ng/ml transforming growth factor β3 (TGFβ3) (Peprotech, London) for 3 weeks[26]. *In vitro *differentiation into chondrocytes was confirmed by morphological changes, Alcian blue staining of differentiated chondrocytes and RT-PCR of Collagen II gene expression in cell homogenate. Total RNA was isolated from the differentiated MSCs using Trizol (Invitrogen, USA). RNA concentrations were measured by absorbance at 260 nm with a spectrophotometer, and 2 μg total RNA was used for reverse transcription using Superscript II reverse transcriptase (Invitrogen, USA). The cDNA was amplified using Taq Platinum (Invitrogen, USA). Osteonectin gene and collagen (II) primers used were designed according to the following oligonucleotide sequence: sense, 5'-GTCTTCTAGCTTCTGGCTCAGC-3'; antisense,5'-GGAGAGCTGCTTCTCCCC-3' (uniGene Rn.133363) and sense, 5'-CCGTGCTTCTCAGAACATCA-3'; antisense, 5'-CTTGCCCCATTCATTTGTCT-3' (UniGene Rn.107239). The RNA templates were amplified at 33 to 45 cycles of 94°C (30 sec), 58°C to 61°C (30 sec), 72°C (1 min), followed with 72°C for 10 min. PCR products were visualised with ethidium bromide on a 3% agarose gel. Glyceraldehyde-3-phosphate dehydrogenase (GAPDH) was detected as housekeeping gene to examine the extracted RNA integrity. CD29 gene expression was also detected by RT-PCR as a marker of MSCs [27].

### Preparation of HCC Model

Hepatocarcinogenesis was induced chemically in rats by injection of a single intraperitoneal dose of diethylnitrosamine at a dose of 200 mg/kg body weight followed by weekly subcutaneous injections of CCl4 at a dose of 3 mL/kg body weight for 6 weeks [28,29]. At the planned time animals were sacrificed by cervical dislocations, blood samples and liver tissues were collected for assessment of the following:

1. Histopathological examination of liver tissues.

2. Gene expressions by qualitative and quantitative real time PCR for the following genes: *β-catenin, PCNA, cyclin D and survivin *genes

3. Alpha fetoprotein by ELISA (provided by Diagnostic Systems Laboratories, Inc., Webstar, Texas, USA.)

### PCR detection of male-derived MSCs

Genomic DNA was prepared from liver tissue homogenate of the rats in each group usingWizard^® ^GenomicDNApurification kit (Promega, Madison, WI, USA). The presence or absence of the sex determination region on the Y chromosome male (sry) gene in recipient female rats was assessed by PCR. Primer sequences for sry gene (forward 5'-CATCGAAGGGTTAAAGTGCCA-3', reverse 5'-ATAGTGTGTAG-GTTGTTGTCC-3') were obtained from published sequences[30,31] and amplified a product of 104 bp. The PCR conditions were as follows: incubation at 94°C for 4 min; 35 cycles of incubation at 94°C for 50 s, 60°C for 30 s, and 72°C for 1 min; with a final incubation at 72°C for 10 min. PCR products were separated using 2% agarose gel electrophoresis and stained with ethidium bromide.

### Labeling stem cells with PKH26

PKH26 is a red fluorochrome. It has excitation (551 nm) and emission (567 nm) characteristics compatible with rhodamine or phycoerythrin detection systems. The linkers are physiologically stable and show little to no toxic side-effects on cell systems. Labeled cells retain both biological and proliferating activity, and are ideal for in vitro cell labeling, in vitro proliferation studies and long term, in vivo cell tracking. In the current work, undifferentiated MSCs cells were labeled with PKH26 according to the manufacturer's recommendations (Sigma, Saint Louis, Missouri, USA). Cells were injected intravenously into rat tail vein. After one month, liver tissue was examined with a fluorescence microscope to detect the cells stained with PKH26. Fluorescence was only detected in the 5th rat group.

### Real-time quantitative analyses for *β-catenin,PCNA,cyclin D and survivin *genes expression

Total RNA was extracted from liver tissue homogenate using RNeasy purification reagent (Qiagen, Valencia, CA). cDNA was generated from 5 μg of total RNA extracted with 1 μl (20 pmol) antisense primer and 0.8 μl superscript AMV reverse transcriptase for 60 min at 37°C. Quantitation of gene expression was conducted using universal probe library sets based real time PCR (Roche diagnostics). Selection of genes specific probes and primers were done using the online ProbeFinder software and the real time PCR design assay of Roche Diagnostics found their website: http://www.universalprobelibrary.com, Hypoxanthine phosphoribosy-ltransferase 1 (Hprt1) was used as a positive control house keeping gene. FastStart Universal Probe Master mix was used in LightCycler^® ^480 Instrument (Roche Applied Science, Indianapolis, USA). Briefly, in the LightCycler^® ^480, a total reaction volume of 20 μl was prepared, of which 2 μl of starting RNA material was included for RT-PCR, a final concentration of 0.5 μM of each forward and reverse primer and 0.2 μM of the TaqMan probe was used. Cycling conditions involve reverse transcription at 50°C for 30 min; enzyme activation at 95°C for 15 min, followed by 50 cycles of 95°C for 10 sec and 60°C for 60 sec. LightCycler^® ^480 RT-PCR data were analyzed using LightCycler1.2 version 3.5 software using the second derivative maximum method. Successfully amplified targets are expressed in Ct values, or the cycle at which the target amplicon is initially detected above background fluorescence levels as determined by the instrument software. Each sample RT-PCR was performed minimally in duplicate, and the mean Ct value with standard deviation reported.

Primer sequences:

1-Beta-Catenin:

- left: acagcactccatcgaccag

- right: ggtcttccgtctccgatct

2-CyclinD:

- left: ttcctgcaatagtgtctcagttg

- right: aaagggctgcagctttgtta

3-PCNA:

- left: gaactttttcacaaaagccactc

- right: gtgtcccatgtcagcaatttt

4-Survivin:

- left: gagcagctggctgcctta

- right: ggcatgtcactcaggtcca

### Analysis of liver Pathology

Liver samples were collected into PBS and fixed overnight in 40 g/Lparaformaldehyde in PBS at 4°C. Serial 5-μm sections of the right lobes of the livers were stained with hematoxylin and eosin (HE) and were examined histopathologically.

## Results

### MSCs culture and identification

**Figure 1:**
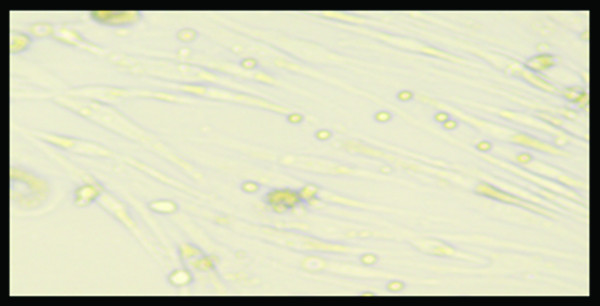
**Undifferentiated mesenchymal stem cells after 2 weeks in culture**. (×20)

**Figure 2:**
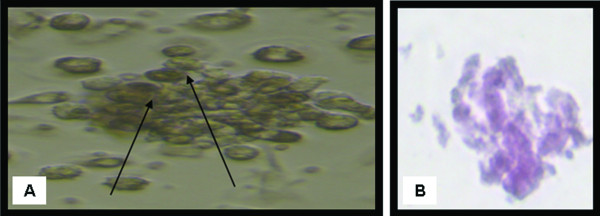
**Morphological and histological staining of differentiated BM-MSCs into osteoblasts**. (A) (×20) Arrows for differentiated MSCs osteoblasts after addition of growth factors. (B) (×200) Differentiated MSCs into osteoblasts stained with Alizarin red stain.

**Figure 3:**
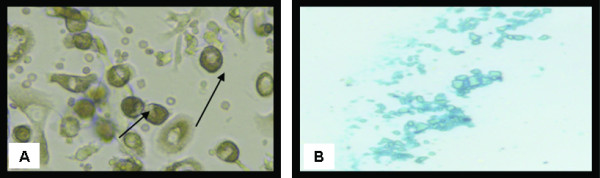
**Morphological and histological staining of differentiated BM-MSCs into chondrocytes**. (A) (×20) Arrows for differentiated MSCs chondrocytes after addition of growth factors. (B) (×200) Differentiated MSCs into chondrocytes stained with Alcian blue stain.

**Figure 4:**
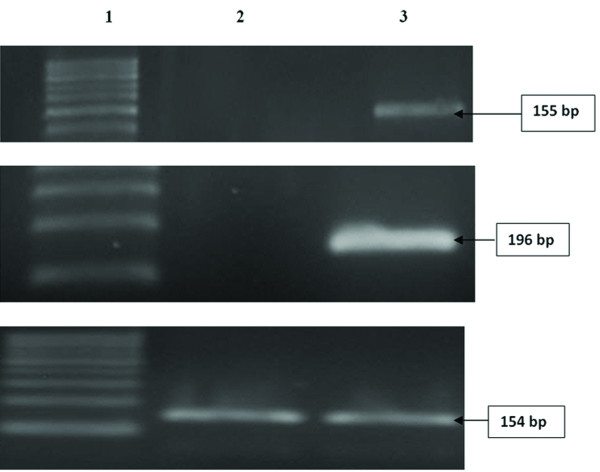
**Agrose gel electrophoresis for Molecular identification of undifferentiated and differentiated BM-MSCs**: (A) gene expression of osteonectin (B) gene expression of collagen II and (C) gene expression of GAPDH in undifferentiated and differentiated MSCs. (A&B) Genes expression of osteonectin and collagen II. Lane 1: DNA marker (100, 200, 300 bp). Lane 2:No PCR product for osteonectin and Collagen II genes in undifferentiated MSCs. Lane 3: PCR product for osteonectin and Collagen II genes in differentiated MSCs (C) Gene expression of GAPDH. Lane 1: DNA marker (100, 200, 300 bp). Lane 2: PCR product for GAPDH gene in undifferentiated MSCs

Isolated and cultured undifferentiated MSCs reached 70-80% confluence at 14 days (Figure 1). In vitro osteogenic and chondrogenic differentiation of MSCs were confirmed by morphological changes and special stains (Figure 2a,b and Figure 3a,b respectively) in addition to gene expression of osteonectin and collagen II (Figure 4a&4b) and GADPH (Figure 4c).

**Figure 5:**
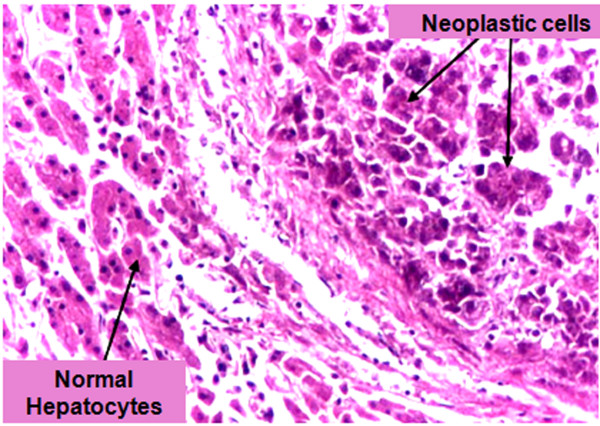
**Hepatocellular carcinoma cells**. (×400) Characterized by large anaplastic carcinoma cells with eosinophilic cytoplasm, large hyperchromatic nuclei and prominent nucleoli. The normal trabecular structure of the liver is distorted.

**Figure 6:**
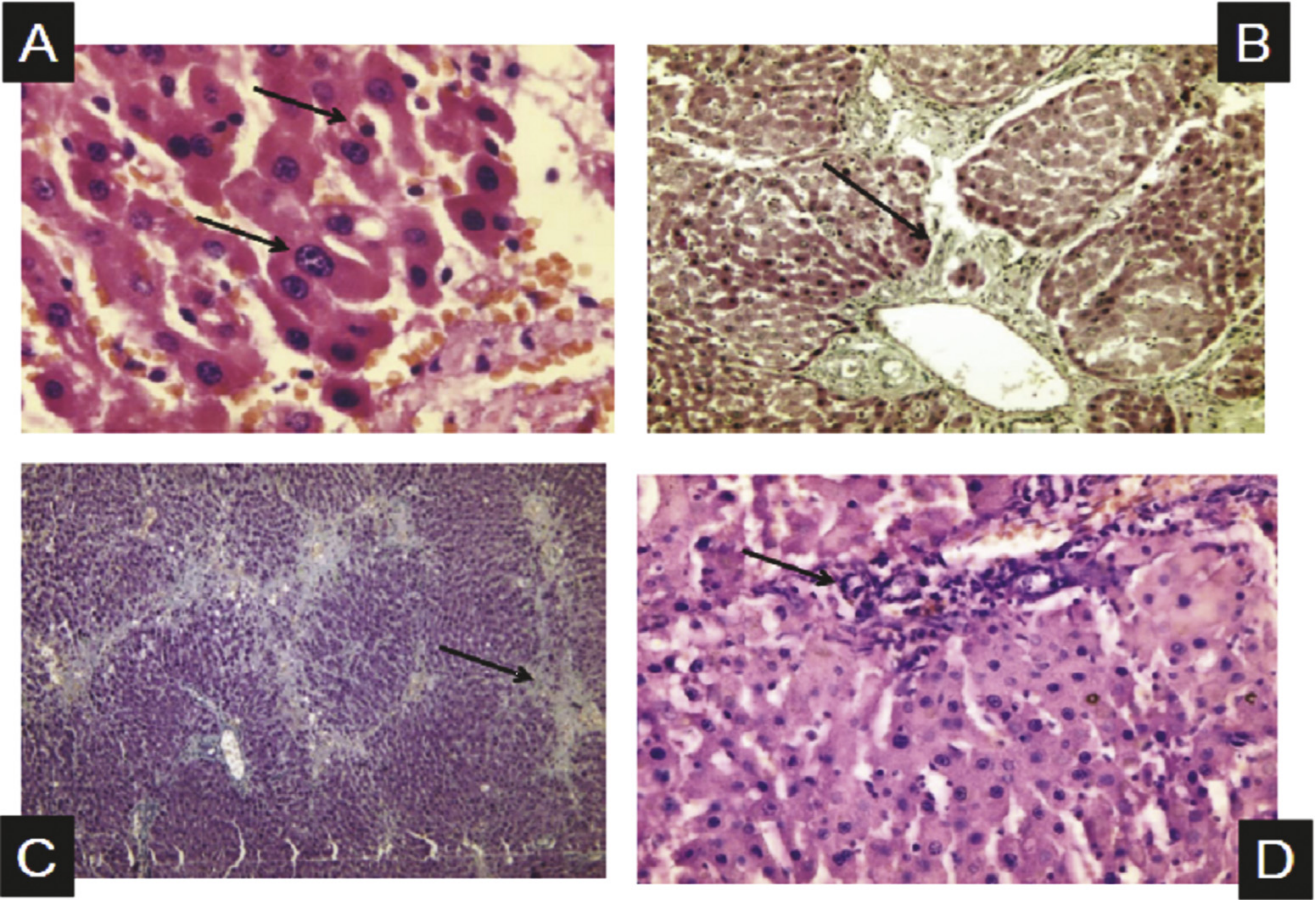
**Histopathological picture of liver tissues in experimental HCC**. Arrows, A: (×400) Small and large cell dysplasia, B: (×200) Macroregenerative nodules type II (borderline nodules) apparent with foci of small cell dysplasia & Increased mononuclear cell infiltrates in portal areas, C: (×200) Focal fatty change & confluent necrosis with active septation, D: (×200) Portal tract showing increased mononuclear cell infiltrates.

**Figure 7:**
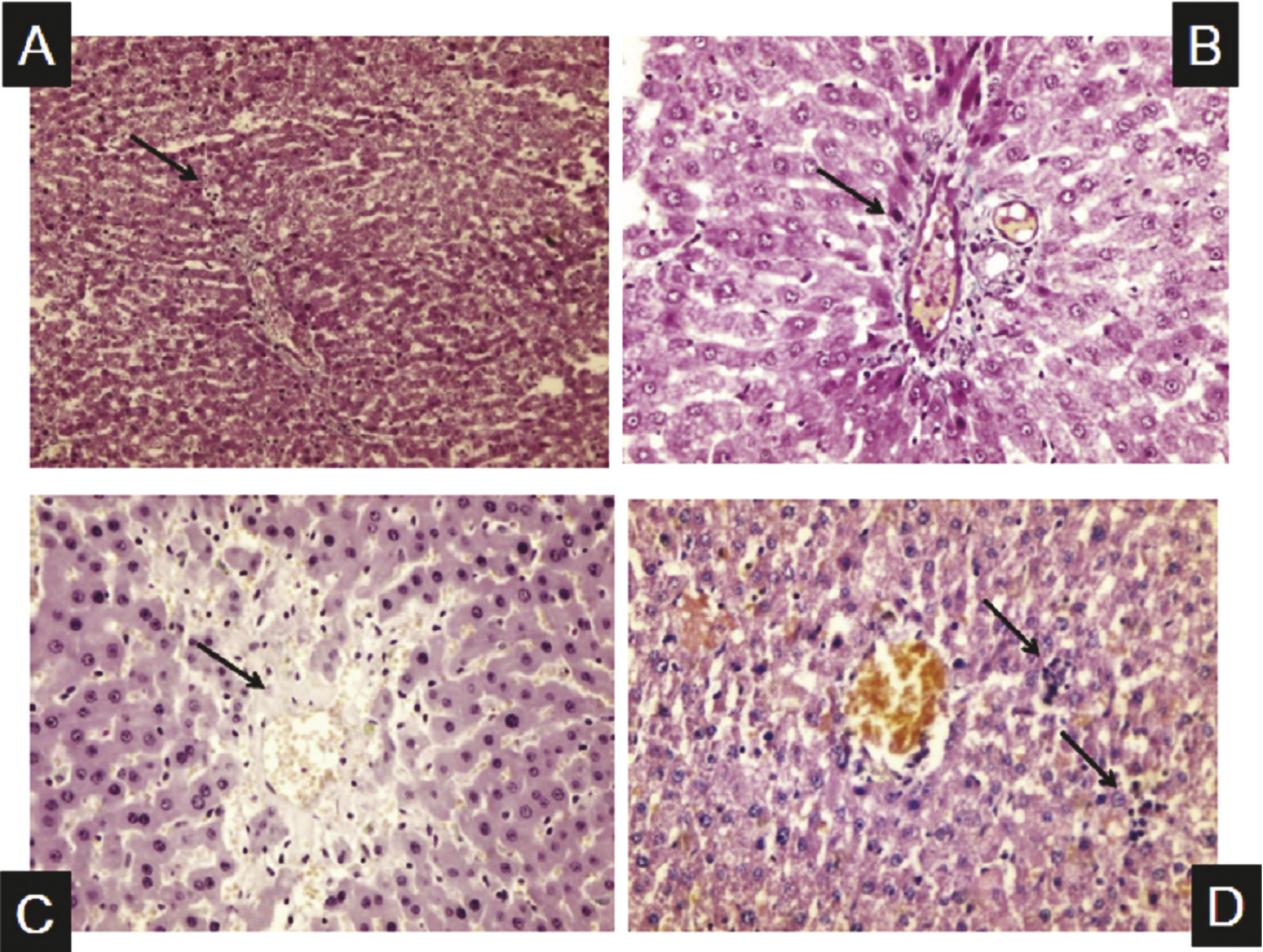
**Histopathological picture of liver tissues in rat that received MSCs after induction of hepatoma**. Arrows, A: (×200) No nodularity & liver cells and lobules appear normal with ballooning degeneration, B: (×400) Normal portal tracts No fibrosis No inflammation, C: (×400) Area of cell drop out with stem cells, D: (×400) No nodularity & liver appears normal, few collections of round to oval stem cells in lobules.

**Figure 8:**
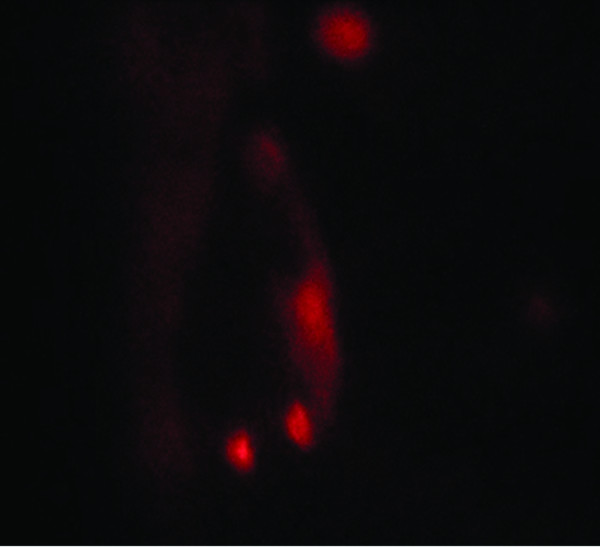
**Detection of MSCs labeled with PKH26 fluorescent dye in liver tissue**. MSCs labeled with the PKH26 showed strong red autofluorescence after transplantation into rats, confirming that these cells were seeded into the liver tissue.

**Figure 9:**
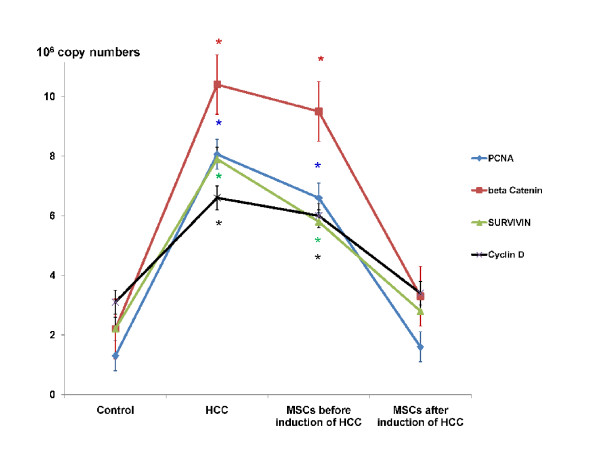
**PCNA, Beta catenin, Survivin and Cyclin D genes expression by real time PCR**. Results are expressed in 10^6 ^copy numbers of each gene mRNA (in 100 ng total RNA). Absolute copy numbers was determined by comparing samples with the standard curve generated. The mRNA level of each gene was normalized with the level of HPRT1 mRNA. * Significant difference in comparison to control (P < 0.05).

**Figure 10:**
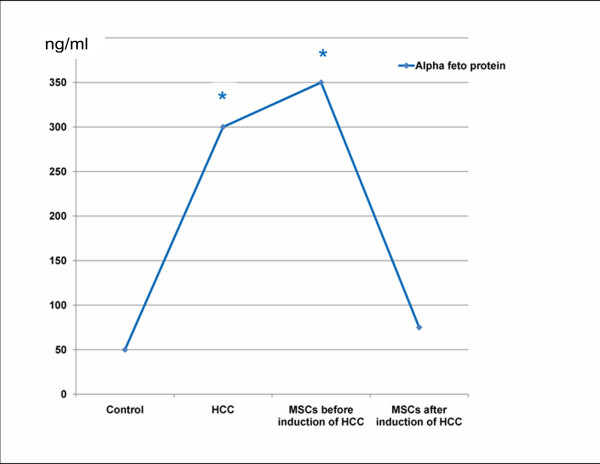
**Alpha fetoprotein levels in ng/ml**. * Significant difference in comparison to control (P < 0.05).

**Figure 11:**
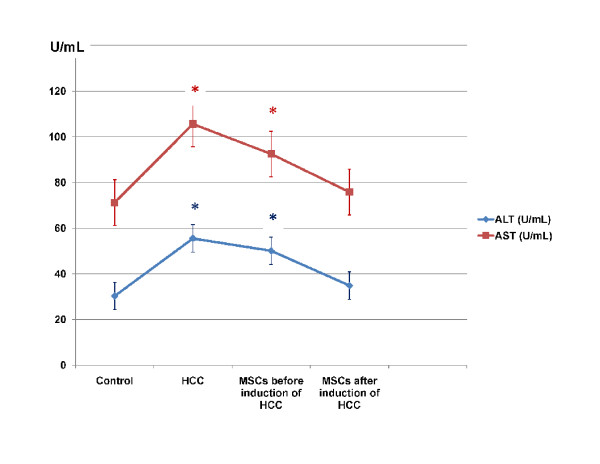
**Serum ALT and AST levels in U/ml**. * Significant difference in comparison to control (P < 0.05).

Histopathology of liver tissues of the animals that received DENA and CCl4 only showed cells with neoplastic changes, anaplastic carcinoma cells, characterized by large cells with eosinophilic cytoplasm, large hyperchromatic nuclei and prominent nucleoli (Figure 5) and macroregenerative nodules typeII (borderline nodules) with foci of large and small cell dysplasia (Figure 6). Improvement of histopathological picture after the administration of MSCs into rats with HCC is demonstrated in figure(7); with minimal reversible liver cell damage in form of ballooning degeneration, areas of cell drop out filled with stem cells, normal areas with sinusoidal dilatation and congestion and absence of fibrous thickening of portal tracts, inflammation, dysplasia and absence of regenerative nodules. Figure (8) shows MSCs labeled with PKH26 fluorescent dye detected in the hepatic tissue, confirming that these cells homed into the liver tissue. Data obtained from the group which received MSCs only and the one which received MSCs solvent were similar to data obtained from healthy controls. On the other hand, HCC rat group and the rat group injected with stem cells prior to induction of HCC (the prophylactic group) showed significant increase in gene expression of all four genes when compared to controls (p < 0.05) (Figure 9), whereas no significant difference in the gene expression was detected in liver tissues of MSCs-treated HCC rats and control group. As regards serum levels of alpha fetoprotein (Figure 10), as well as ALT and AST (Figure 11); significant increase was found in HCC and the prophylactic group(p < 0.05), whereas no significant difference was detected in the HCC rats group treated with MSCs when compared to the control group.

## Discussion

Hepatocellular carcinoma (HCC) is considered as a disease of dysfunction of the stem cells [32]. Stem cells and tumor cells share similar signaling pathways that regulate self-renewal and differentiation, including the Wnt, Notch, Shh and BMP pathways that determine the diverse developmental fates of cells [17-20,33,34]. Therefore, understanding these signaling cascades may provide insights into the molecular mechanisms that underlie stemness and tumorigenesis. In the present study, histopathological examination of liver tissues of the animals group that received DENA and CCl4 was the only one which revealed development of HCC (Figure 1,2). On the other hand, administration of MSCs into rats after induction of experimental HCC led to improvement of histopathological picture with minimal reversible liver cell damage in form of ballooning degeneration, areas of cell drop out filled with stem cells, normal areas with sinusoidal dilatation and congestion and absence of fibrous thickening of portal tracts, inflammation, dysplasia and regenerative nodules. These results reinforce the suggestion of previous studies using animal models which indicated that mesenchymal cells would be more useful for liver regeneration [35-37], as well as the studies which drew attention to the potential of MSCs in regenerative medicine [38].

MSCs were identified by detection of CD29 surface marker, their fusiform shape, adherence, and their ability to differentiate into osteocytes and chondrocytes. Homing of MSCs in liver was confirmed through detection of Y chromosome-containing cells in samples from female recipients of bone marrow cells from male donors, as well as the detection of MSCs labeled with PKH26(Figure 4). Experimental findings in animal models suggest that the induction of parenchymal damage is a prerequisite for successful homing and repopulation with stem cells [39,40]. Molecular mechanisms underlying stem cells mobilization and homing into the injured liver are still poorly understood[41]. However, potential factors and leading pathways have been characterized in these processes, such as the Stromal Cell-Derived Factor-1 (SDF-1)/CXCR4 axis, the proteolytic enzymes matrix metalloproteinases (MMPs), the hepatocyte growth factor (HGF) and the stem cell factor (SCF). The chemokine Stromal Cell-Derived Factor-1 (SDF-1) is a powerful chemo-attractant of hepatic stem cells (HSCs)[42] which plays a major role in the homing, migration, proliferation, differentiation and survival of many cell types of human and murine origin [43]. It is expressed by various bone marrow stromal cell types and epithelial cells in many normal tissues, including the liver [44]. SDF-1 carries on its role through the CXCR4 receptor, a G-protein coupled receptor, expressed on CD34+ hematopoietic stem cells, mononuclear leucocytes and numerous stromal cells [45,46]. Kollet and co-workers [47] also showed that CCl4-induced liver injury (which was the case in the present study)resulted in increased activity of the enzyme MMP-2 and emergence of MMP-9 in the liver of NOD/SCID mice.

As for the mechanisms by which liver regeneration occurs after bone marrow cells transfusion, many mechanisms have been suggested: fusion between hepatocytes and transplanted bone marrow cells has been substantiated as a mechanism by which hepatocytes that carry a bone marrow tag are generated[48], although many studies suggested that cell fusion was not the main mechanism involved in parenchymal repopulation with exogenous cells[49,50]. Another mechanism may be that the stem cells provide cytokines and growth factors in their microenvironment that promote hepatocyte functions by paracrine mechanisms[48]. **Robert and coworkers**[51] stated that the organ microenvironment can modify the response of metastatic tumor cells to therapy and alter the effectiveness of anticancer agents in destroying the tumor cells without producing undesirable toxic effects. In his review, Muraca and coworkers[41] pointed out that, the mechanisms underlying the positive effects reported in preliminary trials are complex and most likely do not involve repopulation of liver parenchyma with bone marrow-derived cells but might result from the production of trophic factors by the infused cells, therefore The identification and characterization of the niche are prerequisites for the identification of stem cells and for understanding their behaviour in physiological and pathological conditions. Niches are local tissue microenvironments that maintain and regulate stem cells [52], **Livraghi and colleagues**[53] stated that the essential role of stem cell microenvironment in preventing carcinogenesis is by providing signals to inhibit proliferation and to promote differentiation. Human MSCs home to sites of Kaposi's sarcoma, and potently inhibit tumor growth *in vivo *by downregulating Akt activity in tumor cells that are cultured with hMSCs prior to transplantation in animal tumor models [54]. Furthermore, tumor cells may secrete proteins that can activate signaling pathways that facilitate MSCs migration to the tumor site. Direct transdifferentiation of cells is another mechanism of liver regeneration, although it has not been demonstrated [48]. However, recent observations shed some light on possible mechanisms underlying the observed bone marrow-derived cells (BMDC) transdifferentiation driven by injured tissues [55]. As a result of injury, tissues release chemokines attracting circulating BMDC, and can produce microvescicles including RNA, proteins and a variety of signals. The authors provided evidence suggesting that these microvescicles are taken up by BMDC and can modify cell phenotype mimicking resident cells in the host tissue. In conclusion, the extensive work performed during the last decade suggests that a series of complex interactions exist between BMDC and injured tissues, including the liver. Microvesicles are mediators of cell reprogramming. Following injury, tissues release chemokines attracting circulating BMDC, and can produce microvesicles including RNA, proteins and a variety of signals. Such microvesicles are taken up by BMDC and can modify cell phenotype mimicking the one of resident cells in the host tissue. Insults trigger the release of chemokines from the endothelium inducing adhesion and migration of circulation BMDC into the liver parenchyma. The liver itself can release powerful signals attracting BMDC and probably contributing to remodeling of their morphology and function. These BMDC in turn can produce molecular signals improving regeneration and function of injured parenchyma. It is to note that, in the present study, administration of MSCs before induction of HCC did not show any tumor suppressive or protective effect. This may be explained by the exposure of MSCs to the chemical carcinogen; DENA and failure of recruitment of MSCs to the liver tissue before exposure to the chemical injury due to the absence of cytokines that recruit MSCs to sites of injury [56]. As regards genetic analysis, results of the present study demonstrated that MSCs downregulated oncogenes expression(Figure 9), where, *β-catenin, PCNA, cyclin D and survivin *genes expression was downregulated in liver tissues of MSCs-treated HCC rats which are all involved in Wnt/β-catenin pathway;one of the main oncogenic pathways involved in HCC[57]. The decreased serum levels of alpha fetoprotein and liver enzymes in the HCC group treated with MSCs indicate the amelioration of the malignant status as well as the liver function of the HCC model.

In recent years, improved knowledge of oncogenic processes and the signaling pathways that regulate tumor cell proliferation, differentiation, angiogenesis, invasion and metastasis has led to the identification of several possible therapeutic targets that have driven the development of molecular targeted therapies. These drugs have showed clinical benefit in patients with various tumor types, including HCC[1].

A major and early carcinogenic event in the development of HCC seems to be the abnormal regulation of the transcription factor β-catenin, a key component of the Wnt signaling pathway [58]. In the normal state, the binding of members of a family of soluble cysteine-rich glycoprotein ligands, the Wnts, to members of the Frizzled family of cell-surface receptors results in the activation of the Wnt signaling pathway. Receptor binding activates DSH (downstream effector Dishevelled), which consequently prevents phosphorylation of β-catenin by glycogen synthase kinase-3β and its subsequent ubiquitination and proteasomal degradation. An ensuing increase in the cytoplasmic concentrations of β-catenin results in its translocation from the cytoplasm to the nucleus. Once in the nucleus, β-catenin acts as a co-activator to stimulate the transcription of genes and expression of gene products involved in cell proliferation (e.g: *c-Myc, Cyclin-D, PCNA*), angiogenesis (e.g: *VEGF*), antiapoptosis (e.g: *Survivin*) and the formation of extracellular matrix [59].

Interestingly, **Schmidt and coworkers**[60] suggested that Iqgap2 acts as a tumor suppressor, and its loss can lead to β-catenin activation and the development of HCC, and this finding further implicates β-catenin as a key driver of HCC. Direct mutation of *β-catenin *is not the only route through which the Wnt pathway can be aberrantly activated in HCC. In their study, **Hoshida and coworkers**[61] stated that, from the three subclasses of HCC that had been characterized, two of them showed either increased Wnt pathway activity or increased MYC/AKT pathway activity. In the present study, overexpression of gene of the Wnt signaling molecule; *β-catenin *and its downstream targets; *PCNA, cyclin D and survivin genes *in liver tissue transformed by DENA, together with their downregulation in MSCs treated rats provids evidence that the Wnt signaling pathway is likely to regulate the inhibitory role of MSCs. Similar suggestions were provided by **Qiao and coworkers**[8]. Also, **Zhu and coworkers**[62] demonstrated that MSCs have an inhibitory effect on tumor proliferation by identifiing that DKK-1 (dickkopf-1) which was secreted by MSCs, acts as a negative regulator of Wnt signaling pathway and is one of the molecules responsible for the inhibitory effect. **Also, Wei and coworkers **studied the inhibition of Wnt-1-mediated signaling as a potential molecular target in HCC and demonstrated that Wnt-1 was highly expressed in human hepatoma cell lines and a subgroup of human HCC tissues compared to paired adjacent non-tumor tissues. An anti-Wnt-1 antibody dose-dependently decreased viability and proliferation of Huh7 and Hep40 cells over-expressing Wnt-1 and harboring wild type *β-catenin*, but did not affect normal hepatocytes with undetectable Wnt-1 expression. Apoptosis was also observed in Huh7 and Hep40 cells after treatment with anti-Wnt-1 antibody. In these two cell lines, the anti-Wnt-1 antibody decreased β-catenin/Tcf4 transcriptional activities, which were associated with down-regulation of the endogenous β-catenin/Tcf4 target genes *c-Myc, cyclin D1*, and *survivin*. They also demonstrated that intratumoral injection of anti-Wnt-1 antibody suppressed *in vivo *tumor growth in a Huh7 xenograft model, which was also associated with apoptosis and reduced c-*Myc,cyclin D1 *and *survivin *expressions [63]. MSCs could upregulate the mRNA expression of cell-cycle negative regulator p21 and apoptosis-associated protease caspase-3, resulting in a G0/G1 phase arrest and apoptotic cell death of tumor cells[64]. They also secrete Dickkopf-1 (DKK-1) to suppress the Wnt/b-catenin signaling pathway, attenuating the malignant phenotype of tumor cells[65].

However, the effect of human bone marrow derived MSCs on the growth of tumoral cells is controversial. HCC was thought to arise from hepatic stem cells; in their study **Ishikawa and colleagues**[66], investigated the malignant potential of hepatic stem cells derived from the bone marrow in a mouse model of chemical hepatocarcinogenesis, their results suggested that hepatic stem cells derived from the bone marrow have low malignant potential, at least in their model.

Regarding their potential therapeutic use in neoplastic diseases, some studies have suggested that adoptively transferred MSCs could favor tumor engraftment and progression *in vivo *[67]. The deleterious effects could derive from different MSCs characteristics. MSCs specifically migrate toward sites of active tumorigenesis, where they could integrate the specialized tumor niche, contribute to the development of tumor-associated fibroblasts and myofibroblasts[68], stimulate angiogenesis[69], and promote the growth and drug resistance of both solid tumors and hematological malignancies[70]. On the contrary, **Secchiero and coworkers**[71] stated that although MSCs release several pro-angiogenic cytokines and promoted the migration of endothelial cells, they found that MSCs when directly cocultured with endothelial cells, significant induction of endothelial cell apoptosis occured. In this respect, their findings are in agreement with those of other authors who have demonstrated that MSCs under certain circumstances might exert anti-angiogenic activity in highly vascularized tumours[72,73], as well as in normal endothelial cell cultures in vitro. **Otsu and coworkers**[73] stated that direct MSCs inoculation into subcutaneous melanomas in an in vivo tumor model, induced apoptosis and abrogated tumor growth. These findings showed for the first time that at high numbers, MSCs are potentially cytotoxic and that when injected locally in tumor tissue they might be effective antiangiogenesis agents suitable for cancer therapy. These controversies can be attributed to many factors such as ratio of MSCs to cancer cells, nature of tumour cells and cancer stem cells, integrity of immune system, number of stem cell passages and site of injection; all can affect the outcome of MSCs use in malignancy. Therefore, the "lack of reproducibility" pointed out by some authorities [74] is at least partially due to large experimental differences in published work. There is thus obvious need for a joined effort by researchers in the field in order to standardize models and procedures both in vitro and in vivo [75].

Several novel findings regarding the role of MSCs in cancer development and/or therapy are summarized from several studies [76,77]: MSCs can behave as potent antigen-presenting cells (APCs) and could be exploited as a new therapeutic tool in cancer therapy in order to amplify immune responses against tumor-specific antigens [12]. **Lu and coworkers**[78] demonstrated that MSCs had potential inhibitory effects on tumor cell growth in vitro and in vivo without host immunosuppression, by inducing apoptotic cell death and G0/G1 phase arrest of cancer cells.

On the basis of the previously reported preclinical data, BM cells seem to facilitate liver regeneration mainly by a microenvironment modulation, which is likely to be transitory. In such a case, multiple treatments would presumably be required to achieve significant and lasting clinical results; technical issues that need to be addressed regard the surface antigens used for MSCs purification, the route of delivery, the amount of infused cells and the timing of infusions[79].

## Conclusions

In conclusion, the present findings demonstrate that MSCs have tumor suppressive effects in chemically induced hepatocarcinogenesis as evidenced by down regulation of Wnt signaling target genes concerned with antiapoptosis, mitogenesis, cell proliferation and cell cycle regulation. Therefore, Wnt signaling might be considered as an important pathway in MSCs-mediated targeting of tumor inhibition. Further studies are recommended regarding the study of different molecular signaling pathways and the precise biologic characteristics of MSCs. Thorough evaluation of MSCs potential risks versus benefits in malignancy still need to be explored.

## Competing interests

The authors declare that they have no competing interests.

## Authors' contributions

MTA, MFE, HA participated in the design of the study and revised it critically; HF, NR, LR, DS, AH, FT carried out the performance the study; SM carried out the analysis of liver pathology; HF, AH performed analysis and interpretation of data and HF, AH drafted the manuscript. All authors read and approved the final manuscript.
